# LINC00974 sponges miR-33a to facilitate cell proliferation, invasion, and EMT of ovarian cancer through HMGB2 upregulation

**DOI:** 10.1590/1678-4685-GMB-2021-0224

**Published:** 2022-01-31

**Authors:** Weiwei Liu, Jing Cheng

**Affiliations:** 1 Maternal and Child Health Hospital of Hubei Province, Department of Gynecology, Wuhan, China; 2 Renmin Hospital of Wuhan University, Department of Obstetrics and Gynecology, Wuhan, China

**Keywords:** LINC00974, miR-33a, HMGB2, ovarian cancer, progression

## Abstract

The function and mechanism of long intergenic non-protein coding RNA 974 (LINC00974) are rarely reported in ovarian cancer (OC). The study aimed to investigate how LINC00974 affects the progression of OC. The expression levels of LINC00974, microRNA-33a (miR-33a), and high mobility group box 2 (HMGB2) mRNA were detected by qRT-PCR. The LINC00974/miR-33a/HMGB2 axis was confirmed by dual-luciferase reporter, RNA-binding protein immunoprecipitation (RIP), and biotinylated RNA pull-down assays. A series of *in vitro* experiments were employed to assess the effects of LINC00974/miR-33a/HMGB2 axis on the proliferation, invasion and epithelial mesenchymal transition (EMT) of OC cells. Results showed that LINC00974 and HMGB2 mRNA expression were upregulated in OC cells, while miR-33a expression was downregulated. HMGB2 was a direct target gene of miR-33a. LINC00974 act as a competing endogenous RNA (ceRNA) to directly bind with miR-33a, thereby upregulated HMGB2 expression. Notably, silencing of LINC00974 suppressed cell proliferation, invasion and EMT of OC cells, whereas miR-33a knockdown partially reversed the phenotypes of LINC00974 on OC cells. Overall, our study demonstrated that LINC00974 sponges miR-33a to promote cell proliferation, invasion, and EMT of OC through HMGB2 upregulation. LINC00974/miR-33a/HMGB2 axis may be an important signaling pathway in the progression of OC.

## Introduction

Ovarian cancer (OC) is one of the most common gynecological malignant tumors and the primary cause of cancer death in women globally (La Vecchia, 2017; Webb and Jordan, 2017). Especially, incidence of OC in young women is increasing obviously in China (Zhang et al., 2019). In the last few decades, despite great advances have been achieved with scientific researches in the therapy of OC, the 5-year overall survival rate of OC patients is less that 40% by global statistics in 2017 (Siegel et al., 2017). Currently, malignant metastasis and cancer recurrence are the main and severe problems in treatment with OC. However, there is a limited understanding of the molecular mechanism underlying the pathogenesis of OC. Therefore, there is an urgent need to seek for effective therapeutic targets to improve the prognosis of this cancer.

Non-coding RNAs (ncRNAs) compose the large majority of the human genomic transcripts (Matsui and Corey, 2017). Long non-coding RNAs (lncRNAs) are a class of ncRNAs of more than 200 nucleotides, which lack the potential to code for protein (Kondo et al., 2017). Recently, studies have shown that dysfunction of lncRNAs plays a pivotal role in biological development and differentiation, and tumorigenesis (Dey et al., 2014; Weidle et al., 2017). Growing evidence has verified that several lncRNAs, such as TPT1 antisense RNA 1 (TPT1-AS1), small nucleolar RNA host gene 12 (SNHG12), associated with poor prognosis of hepatocellular carcinoma (AWPPH), and long intergenic non-protein coding RNA 1127 (LINC01127), can regulate cell proliferation, migration, epithelial mesenchymal transition (EMT), and invasion of human OC cells (Jing et al., 2019; Sun and Fan, 2019; Wu et al., 2019; Yu et al., 2019). As a newly discovered lncRNA, LINC00974 expression is increased in human hepatocellular carcinoma, and serves as an oncogenic factor by promoting proliferation and metastasis (Tang et al., 2014). A previous study in human gastric carcinoma suggested that upregulation of LINC00974 facilitates cell cycle progression (Gao et al., 2019). However, the role of LINC00974 in the progression of OC remains unknown.

MicroRNAs (miRNAs) are a class of conserved non-coding RNA with 18-22 nucleotides and negatively regulate the expression of target genes at the post-transcriptional level (Filipowicz et al., 2008). Evidence has revealed that lncRNAs function as competitive endogenous RNA (ceRNA) to positively regulate target genes of miRNAs (Li et al., 2019). Previously, microRNA-33a (miR-33a) expression was found to be downregulated in human renal cancer and colorectal cancer, and it performed as a tumor suppressive miRNA by targeting mouse double minute 4 (MDM4) and methylenetetrahydrofolate dehydrogenase 2 expression (MTHFD2) (Jiang et al., 2019; Yan et al., 2019). Nevertheless, no literature focuses on miR-33a function in human OC till now. Interestingly, by using bioinformatics analysis, we found that the sequences of LINC00974 transcript and high mobility group box 2 (HMGB2) messenger RNA (mRNA) 3’-non coding region (3’-UTR) were directly bound with miR-33a.

The HMGB2 gene is placed in human chromosome 4q34.1 and encodes a member of the non-histone chromosomal high mobility group protein family, and it is able to efficiently bend DNA and form DNA circles (Thomas, 2001). HMGB2 protein is ubiquitous in various tissues of the human body, and exerts a global genomic role in establishing inactive or active chromatin domains (Taniguchi et al., 2018). There is a study suggested that HMGB2 is associated with malignancy and regulates Warburg effect in human breast cancer by targeting lactate dehydrogenase B and fructose-bisphosphatase 1 (Fu et al., 2018). HMGB2 is expressed in patients with OC and associated with prognosis and tumor metastasis (Ouellet et al., 2006). Based on this, the aim of our study aimed to investigate whether LINC00974 regulates the progression of OC by targeting miR-33a/HMGB2 axis.

## Material and Methods

### Cell selection and cell culture

Three human OC cell lines (SKOV-3, A2780, and OVcAR3) and a normal human ovarian surface epithelial cell line (HOSEpiC) were purchased from the Shanghai Institute of Biochemistry and Cell Biology, Chinese Academy of Sciences (Shanghai, China).The four kinds of cells were grown in RPMI-1640 medium (Thermo Fisher Scientific, Inc., Waltham, MA, USA) containing 10% fetal bovine serum (FBS; Gibco, Grand Island, NY, USA), and incubated with a container contained 5% CO_2_ at 37 °C.

### Quantitative reverse transcription polymerase chain reaction (qRT-PCR)

Using TRIzol reagent (Thermo Fisher Scientific, Inc.,), total RNA was successfully isolated from the cultured cells. The M-MLV reverse transcriptase kit (Takara Biotechnology, Beijing, China) and the PrimeScript RT reagent kit (Takara Biotechnology) were applied to reversely synthesize complementary DNA (cDNA) of each RNA, according to the specification of manufacturer’s protocol. Then cDNA was treated as templates for PCR on ABI 7500 Real-Time PCR system (Applied Biosystems, Foster City, CA, USA) by using the SYBR Premix ExTaq kit (Takara Biotechnology). The thermocycling conditions were as follows: 95˚C for 10 min followed by 40 cycles of 98˚C for 20 s and 60˚C for 45 s. The U6 small nuclear 1 (U6) was applied as an internal control of miR-33a, and the actin beta (ACTB) gene was used as an endogenous control for other coding genes. The primer sequences used in the study were listed in Table 1. Fold change of each gene was quantified by using the 2^-ΔΔcq^ method. The experiment was repeated for three times.

**Table 1 -:** Primer sequences.

Genes	Sequences (5’-3’)
U6	F: CTCGCTTCGGCAGCACA
	R: AACGCTTCACGAATTTGCGT
ACTB	F: TTGTTACAGGAAGTCCCTTGCC
	R: ATGCTATCACCTCCCCTGTGTG
LINC00974	F: GAAGCCGAGCATGAGGAGTT
	R: TGAACTGGCGGTAGGCATTT
miR-33a	F: CCTCATAAGCGGTGCATTGTA
	R: TATGCTTGTTCTCGTCTCTGTGTC
HMGB2	F: GGACCCCAATGCTCCTAA
	R: TGCCCTTGGCACGATATG
VIM	F: CAGGCAAAGCAGGAGTC
	R: TTCAACGGCAAAGTTCTC
ECAD	F: CATTTCCCAACTCCTC
	R: CTTGCCTTCTTTGTCTT
SNAI1	F: CCCCACAGGACTTTGAT
	R: AACCCACGCAGACAGG

U6, U6 small nuclear 1; ACTB, actin beta; LINC00974, long intergenic non-protein coding RNA 974; miR-33a, microRNA-33a; HMGB2, high mobility group box 2; VIM, vimentin; ECAD, E-cadherin, SNAI1, snail family transcriptional repressor 1

### Cell transfection

When cell confluence reached approximately 90%, the cells were subcultured once every two days. After three passages, the cells in logarithmic growth phase were used for cell transfection. The specific short interfering RNA (siRNA) for targeting LINC00974 expression, named si_LINC00974, and the siRNA control (NC) were designed and synthesized at GenePharma Co. Ltd (Shanghai, China). The miR-33a inhibitor and inhibitor control were obtained at Invitrogen corporation from Thermo Fisher Scientific, Inc.. The three kinds of OC cells were transfected with si_LINC00974 and NC or miR-33a inhibitor and inhibitor control at a final concentration of 100 nM by using Lipofectamine® 3000™ transfection reagent (Thermo Fisher Scientific, Inc.,), according to the specification of manufacturer’s instruction. After that, the OC cells were cultured with fresh RPMI-1640 medium containing 10% FBS for 48 h prior to further experiments. 

### Bioinformatics analysis

Bioinformatics software including TargetScanHuman 7.2 (http://www.targetscan.org/vert_72/), miRBase (http://www.mirbase.org/), and microRNA.org (http://www.microrna.org/microrna/home.do), were used to predict the binding sites between miR-33a and HMGB2 mRNA 3’-UTR. Starbase2.0 (http://starbase.sysu.edu.cn/starbase2/index.php) and DIANA-LncBase v2 (http://carolina.imis.athena-innovation.gr/diana_tools/web/index.php?r=lncbasev2/index-predicted) were used to predict LINC00974 transcript that could bind to miR-33a.

### Dual luciferase reporter assay

In order to get the LINC00974 and HMGB2 wild type (WT) reporter plasmids, the sequences of LINC00974 transcript and HMGB2 mRNA 3’-UTR that bound with miR-33a were synthesized and then inserted downstream of the Renilla luciferase reporter gene in psiCHECK™-2 vector (Promega Corporation, Madison, WI, USA). Quick-change site-directed mutagenesis kit (Takara Biotechnology) was applied for direct mutation of miR-33a binding sites in LINC00974 transcript and HMGB2 mRNA 3’-UTR to obtain the LINC00974 and HMGB2 mutant (MUT) reporter plasmids. Then, 2.5 μg of MUT or WT plasmids were co-transfected into OC cells with 100 nM miR-33a inhibitor or inhibitor control by using Lipofectamine® 3000™ transfection reagent. The cells were collected and lysed after 48 h of cell transfection, and the supernatants were obtained by centrifugation at 12,000 x g for 15 min. The dual luciferase detection kit (Promega Corporation) was used for determining the Firefly and Renilla luciferase activities with a TD-20/20 Luminometer (Turner Design, Sunnyvale, CA, USA). Using Firefly luciferase as an internal parameter, the relative Renilla/Firefly activity was normalized and calculated.

### Western blotting analysis

The transfected OC cells were extracted with RIPA lysis buffer (Thermo Fisher Scientific, Inc.,) with 1% protease inhibitor cocktail (Sigma, St Louis, MO, USA). The cellular lysates were obtained by centrifugation at 12, 000g for 30 min at 4°C, and the concentration of protein was quantified with the BCA Protein Assay Kit (Pierce, Rockford, IL, USA). Then, equal amount of proteins (40 μg) was denatured at 105°C for 6 min, and was loaded on 12% sodium dodecyl sulfate-polyacrylamide gel electrophoresis (SDS-PAGE). After transfering onto polyvinylidene fluoride (PVDF; Millipore, Billerica, MA, USA) membranes and blocking with 5% fresh skim-milk (Sigma), the samples were incubated with human HMGB2 (A2973#; 1:1000; ABclonal Technology, Wuhan, China) and ACTB (AC028#; 1:2500; ABclonal Technology) antibodies overnight at 4°C. The ACTB protein was served as a loading control. Subsequently, a secondary HRP-linked second antibody (AS014#; 1:2000; ABclonal Technology) was incubated on the membranes for 1 h at 37°C. Immunoreactive bands were reacted by using enhanced chemiluminescence reaction (Beyotime Biotechnology, Shanghai, China), following standard manufacturer’s instruction.

### Fluorescence microscopy

The cellular localization of LINC00974 was observed by fluorescence microscopy. The enhanced green fluorescent protein plasmid (pEGFP)-LINC00974 overexpressing vector and pEGFP vector were ordered from Hanbio Biotechnology (Shanghai, China). The SKOV-3 cells were transfected with 1.25 μg of pEGFP-LINC00974 overexpressing vector and pEGFP vector by using Lipofectamine® 3000™ transfection reagent. After 24 h cell transfection, the cells were collected for LINC00974 location analysis under a fluorescence microscope. Briefly, the cell were fixed in 4% paraformaldehyde for 15 min at 37°C. After that, the nucleus was counterstained with 1 μl of Hoechst 33258 for 1 min at 37°C (1 mg/ml; Thermo Fisher Scientific, Inc.,), and mounted with Prolong Gold Antifade Reagent (Thermo Fisher Scientific, Inc.,). The image was visualized by using a Laser Scanning Confocal Microscopy (Zeiss, Germany).

### Transwell invasion assay

Cell invasion behavior was evaluated by using a transwell assay. The 24-well transwell chambers (8μm pore size; Millipore) coated with matrigel were prepared, and the transfected OC cells at 4 ×10^4^ cells/ml density was added onto the top chamber with 200 µl serum-free RPMI-1640 medium. Meanwhile, 500 µl RPMI-1640 medium containing 10% FBS was added to the basolateral chamber. After being cultured for 48 h, the cells invaded into the basolateral chamber were treated with 4% paraformaldehyde solution (Sigma) for 20 min at 37°C, followed by crystal violet staining (Beyotime Biotechnology) for 15 min at 37°C. In contrast, the cells in the apical chamber were wiped with cotton swabs. The staining cells were counted for five visual fields (magnification, ×200) with an optical microscope (Olympus, Tokyo, Japan). Each experiment was performed in triplicate.

### Cell counting kit-8 (CCK-8) assay

Cell proliferation ability was assessed by using CCK-8 assay. Briefly, 100 µl RPMI-1640 medium containing 10% FBS was added into 96-well plates, and the transfected OC cells were seeded at the density of 5 × 10^3^/well. 12 µl of CCK-8 reagent (Sigma) was added to each well according to the culture times of 24 h, 48 h, 72 h and 96 h. The optical density (OD) values was measured at the wavelength of 450 nm with a microplate reader (BioTek Instruments, Inc., Winooski, VT, USA). The experiment was repeated for three times.

### RNA-binding protein immunoprecipitation (RIP) assay

RIP assays were performed to confirm the direct interaction between LINC00974 and miR-33a using EZMagna RIP RNA-binding protein immunoprecipitation kit (Millipore), according to the specification of manufacturer’s instruction. Briefly, the OC cells were lysed by using RNA lysis buffer, and then was incubated with magnetic beads conjugated with human anti-argonaute RISC catalytic component 2 (AGO2; 03-110#; Sigma) or IgG (RK00375#; ABclonal Technology) antibody. After incubated overnight at 4 °C, the samples were analyzed by qRT-PCR.

### Biotinylated RNA pull-down assay

The OC cells were transfected with 75 nM biotin labeled NC-bio-probe, WT-bio-miR-33a, and MUT-bio-miR-33a (GenePharma Co.,Ltd). After 48 h, the cells were collected and incubated with lysis buffer (Thermo Fisher Scientific, Inc.,) for 10 min at 4°C. Then, the lysates were treated with streptavidin bead (Millipore) precoated with RNase-free BSA (ABclonal Technology) and yeast tRNA (Thermo Fisher Scientific, Inc.,) overnight at 4°C. After successively washed by precooled pyrolysis buffer, low salt buffer and salt buffer, the bound RNA was purified and analyzed by qRT-PCR.

### Statistical analysis

The data of experiments were presented as the mean ± standard deviation (SD). Statistical analysis was performed by using SPSS 27.0 software (IBM-SPSS, Inc, Chicago, IL, USA). Figures were drawn by using GraphPad Prism 7.0 (San Diego, CA, USA). The comparison between the two groups was performed by Student’s t-test, and the comparison among multiple groups was analyzed by one-way analysis of variance (one-way ANOVA) following Tukey’s post hoc test. *P < 0.05 and **P < 0.01 were considered to indicate a statistically significant difference.

### Availability of data and materials

The datasets during and/or analyzed during the current study are available from the corresponding author on reasonable request.

## Results

### The expression levels of LINC00974, miR-33a, and HMGB2 mRNA in OC cells

**Figure 1 -:**
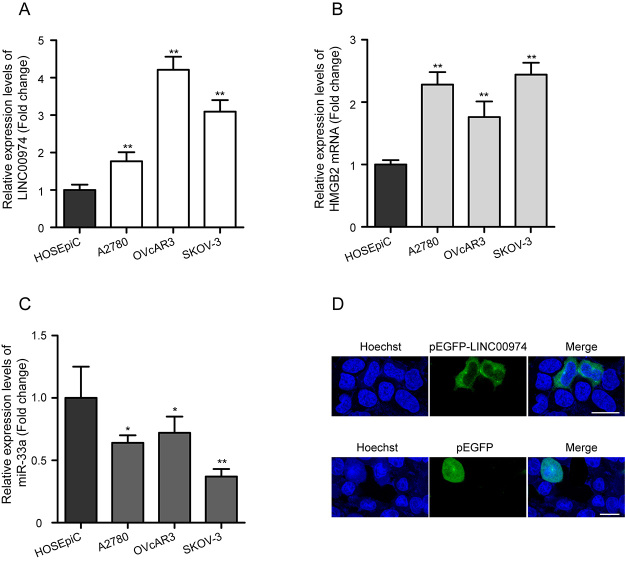
The expression levels of LINC00974, miR-33a, and HMGB2 mRNA in OC cells. **(**A) qRT-PCR analysis of relative expression levels of LINC00974 in SKOV-3, A2780, OVcAR3, and HOSEpiC cells, the ACTB gene was used as an endogenous control. (B) qRT-PCR revealed that the expression level of HMGB2 mRNA was increased in SKOV-3, A2780, and OVcAR3 cells compared with the HOSEpiC cells. (C) Downregulation of miR-33a expression was observed in SKOV-3, A2780, and OVcAR3 cells by qRT-PCR, and the U6 gene was applied as an internal control. (D) Fluorescence microscope analysis of cellular localization of LINC00974 in SKOV-3 cells after transfection of pEGFP-LINC00974 overexpressing vector and pEGFP vector. Scale bar: 10 µm. Data is presented as the mean ± SD of three independent experiments. LINC00974, long intergenic non-protein coding RNA 974; miR-33a, microRNA-33a, HMGB2, high mobility group box 2; mRNA, messenger RNA; OC, ovarian cancer; qRT-PCR, quantitative reverse transcription polymerase chain reaction, HOSEpiC, human ovarian surface epithelial cell line; ACTB, actin beta; U6, U6 small nuclear 1; pEGFP, enhanced green fluorescent protein plasmid; SD, standard deviation. *P < 0.05, **P < 0.01 vs HOSEpiC.

Initially, to identify the role of LINC00974 in the progression of OC, we first assessed its expression in the SKOV-3, A2780, OVcAR3, and HOSEpiC cells. The results of qRT-PCR revealed that LINC00974 levels were higher in the three OC cells than that in the HOSEpiC cells (Figure 1A, P < 0.01), especially in SKOV-3 and OVcAR3. Meanwhile, the expression of HMGB2 mRNA was upregulated in SKOV-3, A2780, and OVcAR3 cells compared with the HOSEpiC cells (Figure 1B, P < 0.01). Interestingly, miR-33a expression was downregulated in SKOV-3, A2780, and OVcAR3 cells compared with the HOSEpiC cells (Figure 1C, P < 0.05). The cellular localization of LINC00974 was investigated by fluorescence microscope. Results showed that LINC00974 was mainly located in the cytoplasm of SKOV-3 cells (Figure 1D), indicating that LINC00974 may exert function by sponging miRNA.

### HMGB2 is a direct target of miR-33a in OC cells

**Figure 2 -:**
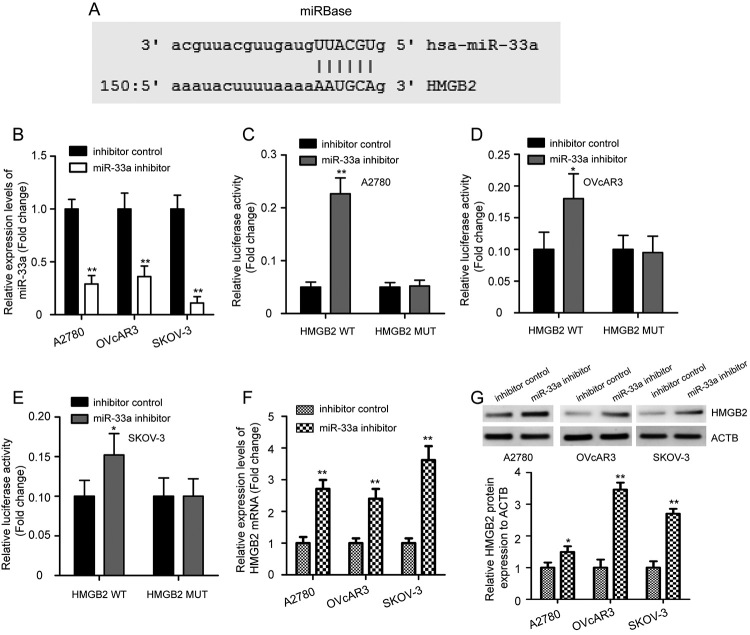
HMGB2 is a direct target of miR-33a in OC cells. (A) Putative miR-33a binding sites in HMGB2 mRNA 3’-UTR by using miRBase. (B) qRT-PCR revealed that miR-33a inhibitor dramatically decreased the expression of miR-33a relative to inhibitor control in OC cells. Dual luciferase reporter assay results following HMGB2 WT or MUT reporter plasmid and miR-33a inhibitor or inhibitor control co-transfection in A2780 (C), OVcAR3 (D), and SKOV-3 (E) cells, and the relative luciferase activity was shown. (F) qRT-PCR analysis of relative expression levels of HMGB2 mRNA in OC cells after transfection of miR-33a inhibitor and inhibitor control. (G) Western blotting analysis indicated miR-33a knockdown elevated HMGB2 protein expression in OC cells. The ACTB protein was served as a loading control. Data were expressed as the mean ± SD (n=3). 3’-UTR, 3’-non coding region; WT, wild type; MUT, mutant. *P < 0.05, **P < 0.01 vs inhibitor control.

HMGB2 mRNA 3’-UTR was predicted to have a potential bind site with miR-33a by using TargetScanHuman 7.2 and miRBase (Figure 2A). To determine whether HMGB2 is a direct target of miR-33a, HMGB2 WT and MUT reporter plasmids were transfected into three OC cells along with the miR-33a inhibitor or inhibitor control, and relative luciferase activity was assessed. In contrast to the inhibitor control group, qRT-PCR validated that the expression levels of miR-33a in the three OC cells was significantly inhibited by miR-33a inhibitor (Figure 2B, P < 0.01). Expectedly, the dual luciferase reporter assay showed that the relative luciferase activity was increased in OC cells co-transfected with miR-33a inhibitor and HMGB2 WT reporter plasmid, whereas this effect was abolished when OC cells were co-transfected with HMGB2 MUT reporter plasmid (Figure 2C-E, P < 0.05). Additionally, HMGB2 mRNA and protein expression levels were also determined in OC cells after treatment with miR-33a inhibitor or inhibitor control. Results revealed significantly elevated HMGB2 expression upon miR-33a knockdown (Figure 2F-G, P < 0.05). The above data indicated that HMGB2 is a direct target of miR-33a in OC cells.

### LINC00974 competitively combines with miR-33a to upregulate expression of HMGB2 in OC cells

**Figure 3 -:**
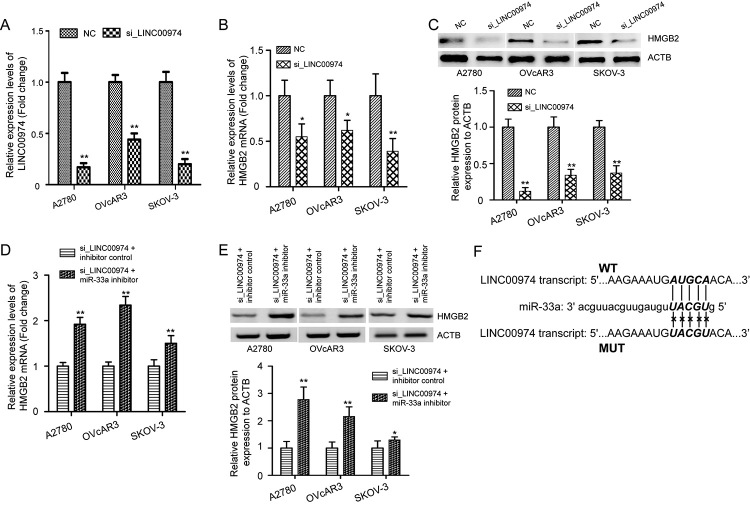
LINC00974 upregulates the expression of HMGB2 in OC cells. (A) qRT-PCR analysis of relative expression levels of LINC00974 in OC cells after transfected with si_LINC00974 or NC. The mRNA (B) and protein (C) expression levels of HMGB2 in si_LINC00974 treated-OC cells were decreased compared with the NC treated-cells by Western blotting analysis. Rescue experiments were performed in OC cells with LINC00974 knockdown by using miR-33a inhibitor, and found that downregulation of HMGB2 mRNA (D) and protein (E) in OC cells caused by inhibiting LINC00974 were reversed by miR-33a inhibitor. (F) Prediction of binding sites between LINC00974 transcript and miR-33a in Starbase2.0 website, and the mutant sites of LINC00974 transcript were shown. si_LINC00974, LINC00974 short interfering RNA; NC, siRNA control. *P < 0.05, **P < 0.01 vs NC or inhibitor control.

To confirm the relationship between LINC00974, miR-33a and HMGB2, we explored the mRNA and protein expression levels of HMGB2 in SKOV-3, A2780, and OVcAR3 cells that treated with si_LINC00974 or NC. qRT-PCR validated that the levels of LINC00974 in OC cells were significantly suppressed by si_LINC00974 (Figure 3A, P < 0.01). Further results showed that LINC00974 silencing notably decreased HMGB2 mRNA and protein levels in OC cells (Figure 3B-C, P < 0.05). Meanwhile, we applied rescue experiments by using miR-33a inhibitor in OC cells with LINC00974 knockdown, and found that si_LINC00974 + miR-33a inhibitor group exhibited higher mRNA and protein expression of HMGB2 than the si_LINC00974 + inhibitor control group (Figure 3D-E, P < 0.05). Starbase2.0 and DIANA-LncBase v2 was performed to predict LINC00974-targeted miR-33a. The complementary sequences between LINC00974 and miR-33a, and the mutant sites of LINC00974 were shown in Figure 3F. 

**Figure 4 -:**
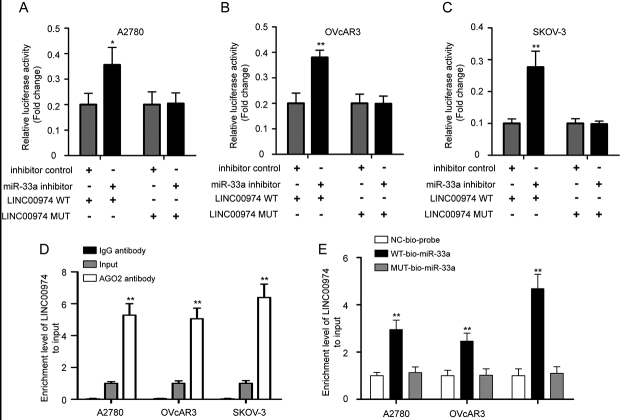
LINC00974 competitively combines with miR-33a in OC cells. The luciferase reporter plasmids containing LINC00974 WT or MUT were co-transfected into A2780 (A), OVcAR3 (B), and SKOV-3 (C) cells with miR-33a inhibitor or inhibitor control, and the relative luciferase activity was calculated. (D) RIP experiments with anti-AGO2 and IgG antibodies were performed in OC cells, and the coprecipitated RNA was used to quantify LINC00974 expression levels by using qRT-PCR. (E) Enrichment of LINC00974 by WT-bio-miR-33a, MUT-bio-miR-33a, and NC-bio-probe was determined by biotinylated RNA pull-down assay. Measurement data were depicted as mean ± SD, repetitions = 3 in each experiment. RIP, RNA-binding protein immunoprecipitation; AGO2, argonaute RISC catalytic component 2. *P < 0.05, **P < 0.01 vs inhibitor control or IgG or MUT-bio-miR-33a.

Subsequently, a dual-luciferase reporter assay confirmed the binding sites between LINC00974 and miR-33a (Figure 4A-C, P < 0.05). Furthermore, the results of RIP assay showed there was higher enrichment of LINC00974 on anti-AGO2 antibody in comparison with IgG antibody (Figure 4D, P < 0.01). Simultaneously, biotinylated the RNA pull-down assay revealed that LINC00974 was abundantly pulled down by WT-bio-miR-33a in comparison with MUT-bio-miR-33a and NC-bio-probe in OC cells, indicating LINC00974 directly bound to miR-33a (Figure 4E, P < 0.01). Based on these data, we confirmed that LINC00974/miR-33a/HMGB2 constructs a ceRNA regulation correlation in OC cells.

### Silencing of LINC00974 suppresses the proliferation, invasion, and EMT of OC cells

**Figure 5 -:**
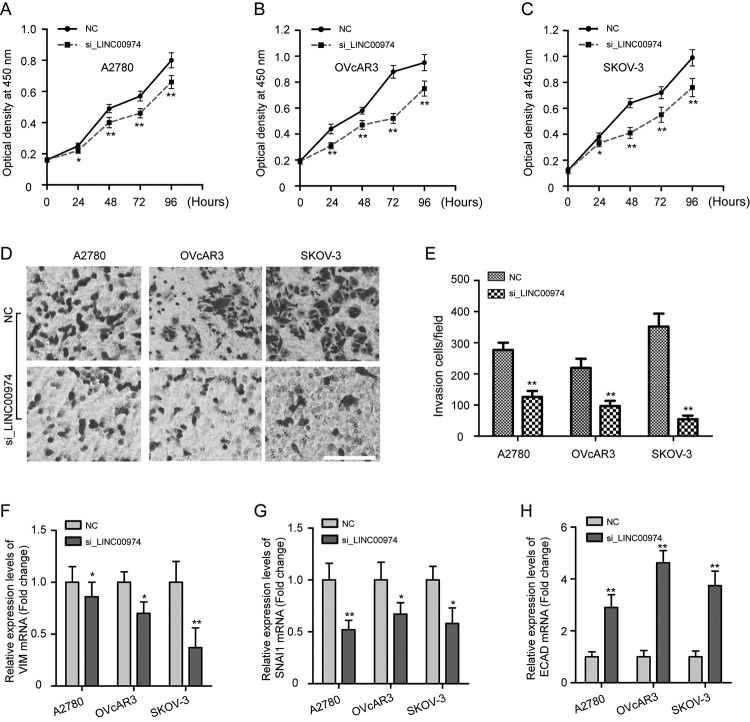
Silencing of LINC00974 suppresses the proliferation, invasion, and EMT of OC cells. Determination of cell proliferation by CCK-8 assay in A2780 (A), OVcAR3 (B), and SKOV-3 (C) cells at 0 h, 24 h, 48 h, 72 h and 96 h times point after treatment with si_LINC00974 and NC. Results showed that knockdown of LINC00974 obviously inhibited the proliferation ability in the three OC cells. (D-E) Transwell invasion assay was applied to observe cell invasion ability after transfection with si_LINC00974 and NC in OC cells. Scale bar: 100 µm. qRT-PCR analysis of relative mRNA expression level of EMT markers including VIM (F), SNAI1 (G), and ECAD (H) in OC cells by LINC00974 knockdown. The experiments were repeated three times, data were presented as the mean ± SD. CCK-8, cell counting kit-8; VIM, vimentin; SNAI1, snail family transcriptional repressor 1; ECAD, E-cadherin. *P < 0.05, **P < 0.01 vs NC.

To determine the effects of LINC00974 on OC cell proliferation, invasion, and EMT, SKOV-3, A2780, and OVcAR3 cells were treated with si_LINC00974 or NC. Noticeably, results of the CCK-8 assay showed that knockdown of LINC00974 inhibited the proliferation ability in the three OC cells (Figure 5A-C, P < 0.05). Similarly, the transwell invasion assay in OC cells was impaired when LINC00974 silencing compared to the NC group (Figure 5D-E, P < 0.01). Additionally, to further insight into the mechanism of LINC00974 on OC cells invasion, the mRNA expression about EMT markers including vimentin (VIM), E-cadheren (ECAD), and snail family transcriptional repressor 1 (SNAI1) was quantified after transfection of si_LINC00974. Expectedly, the expression of VIM and SNAI1 mRNA in OC cells of the si_LINC00974 group was lower than that in NC group (Figure 5F-G, P < 0.05), while ECAD expression was increased (Figure 5H, P < 0.01). These data together indicated that LINC00974 promotes the progression of OC.

### LINC00974 function is partially mediated by regulating miR-33a/HMGB2 axis

**Figure 6 -:**
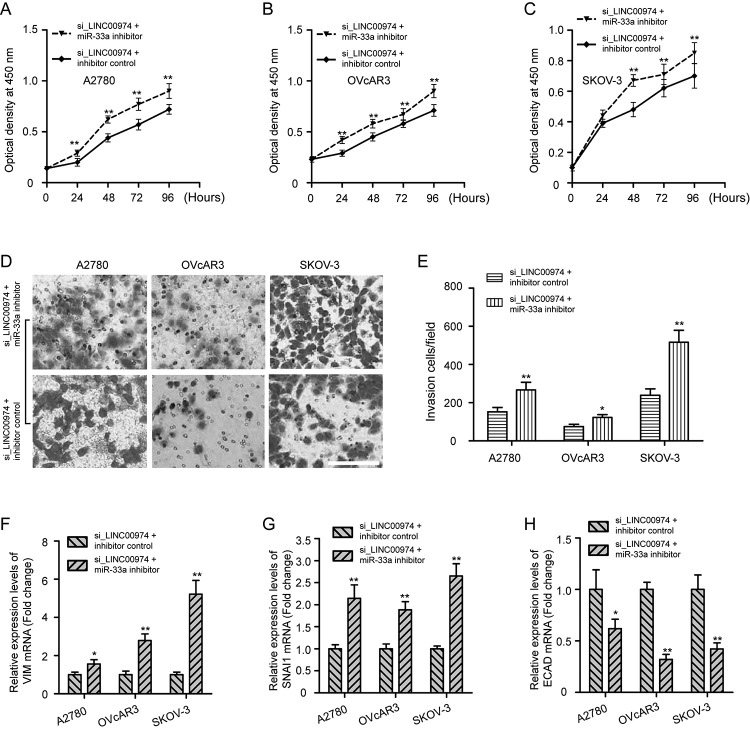
LINC00974 function is partially mediated by regulating miR-33a/HMGB2 axis. (A-C) Rescue experiments were performed in OC cells with LINC00974 knockdown by using miR-33a inhibitor, and found that miR-33a inhibitor promoted the proliferation ability of LINC00974-silenced OC cells. (D-E) Transwell invasion assay indicated inhibition of miR-33a reversed the change in OC cells invasion upon LINC00974 knockdown. Scale bar: 100 µm. (F-H) qRT-PCR analysis of VIM, SNAI1, and ECAD mRNA expression levels in OC cells after cotransfection of si_LINC00974 and miR-33a inhibitor or inhibitor control. Data were shown as mean ± SD of three times. *P < 0.05, **P < 0.01 vs inhibitor control.

Given that LINC00974/miR-33a/HMGB2 constructed a ceRNA regulation correlation, we further focused on whether miR-33a inhibitor reversed the effects of LINC00974 knockdown in OC cells. Data showed that miR-33a inhibitor promoted the proliferation ability of LINC00974-silenced OC cells (Figure 6A-C, P < 0.01). The number of invading cells was also increased by the inhibition of miR-33a and LINC00974 (Figure 6D-E, P < 0.05). In addition, downregulation of VIM and SNAI1 mRNA, and upregulation of ECAD mRNA in OC cells caused by inhibiting LINC00974 were reversed by miR-33a inhibitor (Figure 6F-H, P < 0.05). Therefore, LINC00974 promotes cell proliferation, invasion, and EMT in OC cells through regulation of miR-33a/HMGB2 axis.

## Discussion

An increasing body of evidence has functionally shown that dysregulation of lncRNAs performs both physiological and pathological biological effects in the progression of human OC (Wang et al., 2019). For example, long intergenic non-protein coding RNA 00152 (LINC00152) is shown to be upregulated in OC tissues and regulates the proliferation and cell cycle progression of SKOV-3 cells (Ni et al., 2019). Plasmacytoma variant translocation 1 (PVT1) promotes cell proliferation, migration and invasion through negative regulating miR-133a in OC cells (Yang et al., 2018). Additionally, TP73 antisense RNA 1 (TP73-AS1) promotes ovarian cancer cell proliferation and metastasis via modulation of matrix metallopeptidase 2 (MMP-2) and matrix metallopeptidase 9 (MMP-9) (Wang *et al.*, 2018). In the present study, we aimed to explore the potential role and mechanism of LINC00974 in the progression of OC cells through sponging miR-33a. The obtained results indicated that LINC00974 promotes cell proliferation, invasion, and EMT in OC cells via upregulating the expression of HMGB2 by competitive binding to miR-33a.

Recently, LINC00974 expression has been reported to be increased in hepatocellular carcinoma, gastric carcinoma, and oral squamous cell carcinoma (Tang *et al.*, 2014; Gao *et al.*, 2019; Tian et al., 2021). Besides, elevated LINC00974 expression is associated with oral fibrogenesis through activating TGF-β/Smad signaling pathway (Fang et al., 2019). Similarly, upregulated LINC00974 expression was found in SKOV-3, A2780, and OVcAR3 cells in our study. Furthermore, we transfected si_LINC00974 and NC into OC cells to determine the role of LINC00974. Function studies revealed that silencing of LINC00974 suppressed proliferation, invasion, and EMT of OC cells. Consistent with our study, evidence has confirmed that LINC00974 acts as an oncogenic factor to accelerate proliferation and metastasis in hepatocellular carcinoma (Tang *et al*., 2014). LINC00974 downregulates miR-122 to upregulate RhoA in oral squamous cell carcinoma, thereby promoting cell invasion and migration (Tian *et al*., 2021). 

Increasing studies have widely proved that lncRNAs regulate cancer cell biological properties through sponging with target miRNAs, thereby preventing the miRNAs by binding to their target genes (Zhou et al., 2017). The role of lncRNAs is closely related to their cellular localization. In our study, we found that LINC009741 was mainly localized to the cytoplasm, suggesting that LINC009741 might function as an endogenous miRNA sponge. miR-33a has been reported as a antitumor miRNA in a variety of human cancers including gallbladder cancer, non-small-cell lung carcinoma, osteosarcoma, and prostate cancer (Zhang et al., 2016; Du et al., 2017; Karatas et al., 2017; Huang et al., 2018). Meanwhile, cancer susceptibility candidate 15 (CASC15) promotes cell EMT and facilitates malignancy of hepatocellular carcinoma cells via miR-33a sponging (Li et al., 2019). In glioma, differentiation antagonizing non-protein coding RNA (DANCR) facilitates tumor malignancy by sponging miR-33a (Yang et al., 2018). Our study revealed that LINC00974 directly bound to miR-33a through a luciferase assay. RIP assay further verified that LINC00974 was enriched by anti-AGO2 antibody, and biotinylated RNA pull-down assay revealed that LINC00974 was pulled down by miR-33a. These results suggested that LINC00974 acts as a ceRNA to directly bind with miR-33a in OC cells. 

Finally, we used bioinformatics analysis to predict miR-33a target genes, followed by validation using a dual luciferase reporter assay. We found that HMGB2 was directly regulated by miR-33a, and the expression of HMGB2 in OC cells was modulated by LINC00974. HMGB2 is a member of the HMGB protein family, which is widely expressed in human tissues and comprises ubiquitous, abundant nonhistone nuclear proteins with diverse functions (Taniguchi *et al*., 2018). Evidence by Li *et al.* demonstrated that HMGB2 acts as a tumor promoter in OC by promoting cell growth and metastasis of ovarian cancer cells (Li *et al.*, 2018). In our study, the phenotypic change of OC cells by si_LINC00974 was similar with HMGB2 down-expressing by Li et al. Therefore, we assumed that LINC00974 could upregulate HMGB2 expression by competitively sponging miR-33a, thereby enhancing cell proliferation, invasion, and EMT of OC cells. This was confirmed by a series of rescue experiments wherein miR-33a inhibition reversed LINC00974 knockdown-induced downregulation of HMGB2 expression. More importantly, miR-33a knockdown also overcame the effects of LINC00974 silencing on OC proliferation, invasion, and EMT, suggesting a oncogenic role for LINC00974 in regulating OC progression by regulating miR-33a/HMGB2 axis. Nevertheless, the mechanism network of LINC00974 in regulating OC progression is still not clear. Thus, further understanding the regulatory mechanism of multiple molecules in OC progression is needed.

## Conclusion

The present study demonstrated that LINC00974 expression is upregulated in OC cells. Notably, LINC00974 sponges miR-33a to promote cell proliferation, invasion, and EMT of OC cells through HMGB2 upregulation, which indicated that LINC00974/miR-33a/HMGB2 axis may be an important signaling pathway in the progression of OC.
